# Differential Development of Glucose Intolerance and Pancreatic Islet Adaptation in Multiple Diet Induced Obesity Models

**DOI:** 10.3390/nu4101367

**Published:** 2012-09-28

**Authors:** Bilal Omar, Giovanni Pacini, Bo Ahrén

**Affiliations:** 1 Department of Clinical Sciences, Medicine, Lund University, SE221 84, Lund, Sweden; Email: .BoAhren@med.lu.se; 2 Metabolic Unit, Institute of Biomedical Engineering, National Research Council, 35127, Padova, Italy; Email: giovanni.pacini@isib.cnr.it

**Keywords:** insulin sensitivity, beta cell adaptation, insulin secretion, insulin resistance, diabetes, high-fat diet, mouse

## Abstract

*Background*: The C57BL/6 mouse fed a high fat diet is a common and valuable model in experimental studies of obesity and type 2 diabetes (T2D). Different high fat diets are used and in order to determine which diet produces a model most accurately resembling human T2D, they need to be compared head-to-head. *Methods*: Four different diets, the 60% high fat diet (HFD) and the 58% high fat-high sucrose Surwit diet (HFHS) and their respective controls, were compared in C57BL/6J mice using glucose tolerance tests (IVGTT) and the euglycemic clamp. *Results*: Mice fed a HFD gained more weight than HFHS fed mice despite having similar energy intake. Both high fat diet models were glucose intolerant after eight weeks. Mice fed the HFD had elevated basal insulin, which was not seen in the HFHS group. The acute insulin response (AIR) was unchanged in the HFD group, but slightly increased in the HFHS diet group. The HFHS diet group had a threefold greater total insulin secretion during the IVGTT compared to its control, while no differences were seen in the HFD group. Insulin sensitivity was decreased fourfold in the HFD group, but not in the HFHS diet group. *Conclusion*: The HFD and HFHS diet models show differential effects on the development of insulin resistance and beta cell adaptation. These discrepancies are important to acknowledge in order to select the appropriate diet for specific studies.

## 1. Introduction

Obesity and diseases associated with it, represent a rapidly growing health problem in both developed and developing countries [1]. Type 2 diabetes (T2D) is the most prevalent co-morbidity of obesity [2]. There are clear biochemical relations between obesity and T2D, where changes in obesity predispose susceptible individuals to develop T2D. An important mechanism is that the expansion of adipose tissue depots that occurs in obesity leads to elevated levels of adipocyte-derived hormones, circulating free fatty acids and pro-inflammatory cytokines. Some of these factors contribute to peripheral insulin resistance in the liver, adipose tissue and skeletal muscle [3]. The insulin resistance in turn would cause impaired glucose tolerance, however most individuals are able to adapt to the insulin resistance through a compensatory increase in insulin secretion resulting in normal circulating glucose levels. Adaptation to the insulin resistance by increasing insulin secretion reaches a critical point in some individuals where they can no longer maintain insulin secretion at such high levels [4]. Pancreatic beta cell function becomes at this point insufficient resulting in fasting and postprandial hyperglycemia and eventually type 2 diabetes. It is known that reduced beta cell volume and function are associated with the insulin insufficiency seen in impaired glucose tolerance and T2D [4,5]. With the reduced beta cell volume and function, the counter-regulation of glucagon secretion is lost resulting in fasting and postprandial hyperglucagonemia which contributes to the existing hyperglycemia [6]. As islet adaptation to insulin resistance is critical to preventing the onset of T2D, treatment strategies that involve restoring or maintaining beta cell function are currently in use and new treatments are actively being pursued.

In the development of new treatments for T2D the necessity for reliable animal models is of the utmost importance. The C57BL/6 mouse strain has been shown to become obese, insulin resistant and glucose intolerant in response to consumption of a high (58%–60%) fat diet and is the most used model for studying diet induced obesity and its co-morbidities [7,8]. In response to the high fat diet C57BL/6 mice show an initial impairment of glucose tolerance and insulin secretion [9]. However after several weeks, high fat fed mice show increases in their acute insulin response to a glucose challenge and improved glucose disposal resulting in a near normalization of glucose tolerance [9]. This adaptation to insulin resistance is due to the remarkable capacity for C57BL/6 mice to increase beta cell mass and volume in response to high fat feeding [10,11]. The increased beta cell mass and insulin secretion result in higher fasting and postprandial insulin levels and normalized glycemia [10,11]. The ability of C57BL/6 mice to adapt to insulin resistance by increasing insulin secretion is similar to the adaptation to insulin resistance seen in humans [12]. There are however key differences that limit the high fat diet fed mouse model in the study of diabetes. In mice fed a high fat diet for 1 year, beta cell volume increases 2–3 fold [10,11]. In human obesity, more modest increases in beta cell volume from 20% to 50% have been reported [13,14]. Some obese insulin resistant humans will experience beta cell failure and insulin levels will no longer be sufficient to maintain normal glycemia. Once this process begins, fasting and post-prandial hyperglycemia and eventually overt diabetes is the result. Mice fed a high fat diet do not develop the progressive beta cell failure seen in human diabetes [8], so diet induced obesity mouse models do not develop overt T2D as is the case in the human disease. 

Metabolic studies performed in rodents from different research labs have yielded some variable results. There are many reasons why this may be the case, such as gender, age, mouse substrain, use of anesthetics among others. Whether or not the different high fat diets used to induce obesity and insulin resistance in mice contributes to this variation may also be discussed. Other diets which are proposed to more closely mimic human diets have been described [15,16,17]. Although all of the diets are reported to induce obesity, the amount and source (animal or vegetable) of fat and the composition of other macronutrients are different in different diets. It is likely that differences in the type and composition of different macronutrients in the diets could induce glucose intolerance and/or beta cell dysfunction in different manners, although this has not been investigated thoroughly. In order to determine which dietary regime results in the most appropriate mouse model for studies on obesity, glucose intolerance and islet adaptation to insulin resistance, the diets need to be evaluated in head to head comparisons. This study describes a head to head comparison of the two most commonly utilized diet induced obesity models for the study of obesity and type 2 diabetes, with a focus on the increased insulin secretion in response to insulin resistance as the primary endpoint.

## 2. Materials and Methods

### 2.1. Animals and Diet Regimes

Six week old female C57BL6/J BomTac mice were obtained from Taconic, Skensved, Denmark, and kept in a temperature controlled room (22 °C) on a 12-h light-dark cycle, with food and water ad libitum. On arrival, all mice were placed on a normal rodent diet (ND) D12450B for 1 week to acclimate to hard feed and thereafter divided into four groups and fed with ND, high fat diet (HFD) D12492, Surwit high sucrose control diet (HS) D12329G or Surwit high fat-high sucrose diet (HFHS) D12331; all diets were from Research Diets, New Brunswick, NJ; for compositions see Table 1 and Table 2. Body weight and food consumption was measured weekly. This study was approved by the regional animal ethical committee, Lund, Sweden.

**Table 1:** Dietarycomposition of the experimental diets.

Diet (kcal%)	ND	HFD	HS	HFHS
**Protein**	20	20	16.4	16.4
**Carbohydrate**	70	20	73.1	25.5
**% from sucrose**	34.5	6.7	60	12.6
**Fat**	10	60	10.5	58
**% Saturated **	2.4	19.2	7.0	54.1
**% Monounsaturated**	3.0	21.5	1.0	1.4
**% Polyunsaturated**	4.7	19.2	2.5	2.5
**% from lard**	44	91	0	0
**% from coconut/soybean oil**	56	9	100	100

All values in the table, with the exception of fat source (lard, coconut/soybean oil), represent the percentage of total kcal in the respective diets derived from the indicated macronutrient. The values for the fat source represent the percentage of the total kcal from fat derived from the indicated source. ND—Normal Diet, HFD—High Fat Diet, HS—High Sucrose Diet, HFHS—High Fat-High Sucrose Diet.

**Table 2:** Carbohydrate and Fatty acid compositions of the experimental diets.

*All values as % weight*	ND	HFD	HS	HFHS
**Carbohydrate Composition**				
**Corn Starch**	42	0	0	0
**Maltodextrin 10**	5	51	17	49
**Sucrose**	47	28	83	51
**Cellulose**	7	21	0	0
**TOTAL**	100	100	100	100
**Fatty Acid Composition**				
**C2, Acetic**	0	0	0	0
**C4, Butyric **	0	0	0	0
**C6, Caproic **	0	0	0.4	0.6
**C8, Caprylic **	0	0	4.8	7.2
**C10, Capric **	0	0.0	3.6	5.5
**C12, Lauric **	0	0.1	29.3	44.3
**C14, Myristic **	0.5	1.1	11.2	16.8
**C14:1, Myristoleic **	0	0	0	0
**C15 **	0	0.1	0	0
**C16, Palmitic **	14.9	19.6	9.3	8.8
**C16:1, Palmitoleic **	0.7	1.3	0	0
**C17 **	0.2	0.4	0	0
**C18, Stearic **	7.1	10.6	8	10.1
**C18:1, Oleic **	28.8	34.1	9.9	2.4
**C18:2, Linoleic ω-6**	41.9	28.8	20.6	3.8
**C18:3, Linolenic ω-3**	5.0	2.0	2.9	0.6
**C18:4, Stearidonic **	0	0	0	0
**C20, Arachidic **	0	0.2	0	0
**C20:1 **	0.2	0.6	0	0
**C20:2 **	0.5	0.8	0	0
**C20:3 **	0	0.1	0	0
**C20:4, Arachidonic **	0.2	0.3	0	0
**TOTAL**	100	100	100	100
**Saturated (%)**	22.7	32.0	66.5	93.2
**Monounsaturated (%)**	29.7	36.0	9.9	2.4
**Polyunsaturated (%)**	47.6	32.0	23.6	4.3
**PUFA:MUFA**	0.6	1.1	0.4	0.6
**UFA:SFA**	3.4	2.1	0.5	0.1
**Short chain fatty acids (%)**	0	0	0	0
**Medium chain fatty acids (%)**	0	0.1	38.1	57.5
**Long chain fatty acids (%)**	100	100	62	42
***n*-6 fatty acid (%)**	41.9	28.8	20.6	3.8
***n*-3 fatty acid (%)**	5.0	2.0	2.9	0.6

All values in the table represent the percentage of the total dry weight of the diet for the indicated carbohydrate or fatty acid. ND—Normal Diet, HFD—High Fat Diet, HS—High Sucrose Diet, HFHS—High Fat-High Sucrose Diet.

### 2.2. Intravenous Glucose Tolerance Tests

An Intravenous glucose tolerance test (IVGTT) was performed on 5-h fasted mice after 8 weeks of diet treatment. Blood samples were collected from anaesthetized mice ((Hypnorm, 0.5 mg fluanisone, 0.02 mg fentanyl per mouse; Janssen, Beerse, Belgium), (0.25 mg midazolam per mouse; Dormicum, Hoffman-LaRoche, Basel, Switzerland)) from the retrobulbar, intraorbital, capillary plexus before D-glucose administration (0.35 mg/kg bw) to the tail vein. Additional blood samples were collected at 1, 5, 10, 20, 50 and 75 min from each mouse. Plasma was separated and stored at −20 °C until it was analyzed for insulin and glucose.

### 2.3. Hyperinsulinemic-Euglycemic Clamp

Hyperinsulinemic-euglycemic clamp studies were performed on anesthetized C57BL6/J BomTac mice after 8 weeks on their respective diets. The clamp experiments were carried out as previously described [18]. Briefly, before the experiment, the right jugular vein (infusion) and the right carotid artery (blood sampling) were catheterized. The mice remained anesthetized to reduce variation in the glucose concentrations due to stress from the restraints. Thirty minutes after the introduction of the catheters synthetic human insulin (Actrapid, Novo Nordisk, Bagsvaerd, Denmark) was infused as a primed (40 mU) continuous (15 mU·kg^−1^·min^−1^) infusion. The volume load was 4 μL for the first minute and 2 μL/min thereafter. Blood glucose (5 μL whole blood) was determined every 5 min with an Accu-Chek Aviva blood glucose monitor (Hoffman-LaRoche, Basel, Switzerland). A variable rate of glucose (solution of 20–30 mg/dL) was infused to maintain blood glucose levels at 5.5–6.5 mmol/L. Glucose disappearance was represented by the average glucose infusion rate during the steady state (final 30 min of the clamp).

### 2.4. Statistical Analysis

All data are presented as mean ± SEM. The acute insulin response (AIR) was calculated as the mean of the suprabasal 1 and 5 min values, while ΔAIR was calculated as the mean of the suprabasal 1 min value only. The glucose elimination constant (KG) was calculated as the slope of the logarithmically transformed circulating glucose concentration between 5 and 20 min after administration of the glucose bolus. Metabolic efficiency was assessed as energy intake (kJ) per weight gained (g), whereas feed efficiency was assessed with the equation ((weight gain (g)/energy consumed (kcal)) × 100). Mice were not housed individually so data was taken per cage. In both cases the energy intake was the total energy intake for each cage of mice and weight gain was determined as the sum of the weight gained by all the mice in a given cage. Comparisons between the experimental and control groups were performed by the Mann-Whitney *U*-test. Differences were considered statistically significant when *p *< 0.05.

## 3. Results

**Figure 1:**
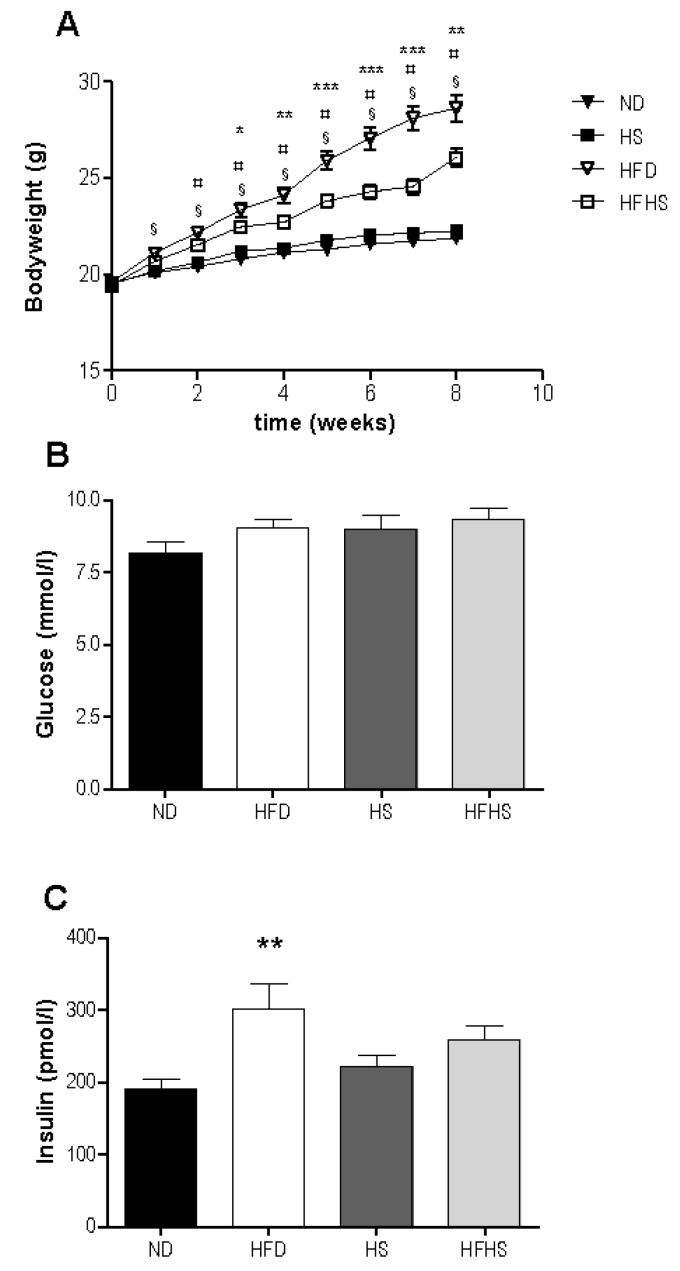
Weight gain and basal blood glucose and insulin, (**A**) Bodyweight was measured once weekly for the 8 week study period. *n *= 34 per diet group, ^§^* p* < 0.001 normal rodent diet (ND) *vs.* high fat diet (HFD), ^¤^* p* < 0.01 high sucrose control diet (HS) *vs.* high fat-high sucrose Surwit diet (HFHS), * *p* < 0.05, ** *p* < 0.01, *** *p* < 0.001 HFD *vs.* HFHS. Blood samples were taken from the retrobulbar intraorbital plexus after a 5 h fast and plasma was assayed for glucose (**B**) and insulin (**C**). *n* = 11–12 per diet group, ** *p* < 0.01.

There was an increase in bodyweight in response to high fat feeding in both groups with significant differences between the high fat diet groups and their respective low fat controls evident after only two weeks (Figure 1A). The high fat diet fed group gained significantly more weight than did the high fat-high sucrose diet group, despite consuming nearly identical amounts of energy (2695 ± 88 kJ *vs.* 2646 ± 153 kJ). Food consumption was not significantly different between the diet groups (Table 2). Metabolic efficiency was lower in both high fat diet groups than in their respective normal diet controls, but there were no significant differences between high fat diet groups (Table 3). Feed efficiency was greater in both high fat diet models compared to their respective controls. In addition high fat diet fed mice had significantly higher feed efficiency than the high fat-high sucrose diet fed mice (Table 3). 

Basal glucose levels were not significantly different between any of the diet groups (Figure 1B). Basal insulin levels were significantly higher in the high fat diet group than in the normal diet group, but not significantly different between the high sucrose control and high fat-high sucrose diet groups (Figure 1C). 

**Table 3:** Feed consumption, metabolic efficiency and feed efficiency.

Feed Consumption	ND	HFD	HS	HFHS
**g/mouse**	132 ± 3	123 ± 4	133 ± 5	114 ± 7 ^b,c^
**kcal/mouse**	509 ± 13	644 ± 21 ^a^	542 ± 20	632 ± 36
**kJ/mouse**	2132 ± 55	2695 ± 87 ^a^	2268 ± 86	2646 ± 153
**Weight gain (g)**	2.2 ± 0.4	9.5 ± 0.8 ^a^	2.8 ± 0.2	6.7 ± 0.4 ^b^
**Metabolic efficiency (kJ/g)**	869 ± 91	291 ± 20 ^a^	767 ± 25	407 ± 51 ^b^
**Feed efficiency (g/kcal)**	0.4 ± 0.1	1.5 ± 0.1 ^a^	0.5 ± 0.03	1.1 ± 0.1 ^b,c^

^a^ *p* < 0.05, ND *vs.* HFD; ^b^* p* < 0.05, HS *vs.* HFHS; ^c^* p* < 0.05, HFD *vs.* HFHS. ND—Normal Diet, HFD—High Fat Diet, HS—High Sucrose Diet, HFHS—High Fat-High Sucrose Diet.

**Figure 2:**
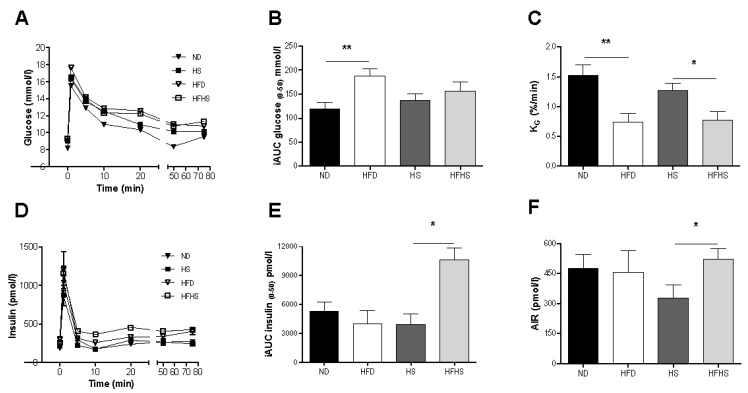
Intravenous glucose tolerance tests (IVGTT). 0.35g/kg bw D-glucose was injected into the tail vein and blood was sampled from the retrobulbar intraorbital plexus at 0, 1, 5, 10, 20, 50 and 75 min. (**A**) Plasma glucose concentrations during the IVGTT. (**B**) Incremental area under the curve for glucose from 0 to 50 min after the glucose injection. (**C**) The glucose elimination constant KG was determined as the rate of change in the logarithmic glucose concentration between 5 and 20 min after the glucose injection. (**D**) Plasma insulin concentrations during the IVGTT. (**E**) Incremental area under the curve (AUC) for insulin from 0 to 50 min after the glucose injection. (**F**) The acute insulin response (AIR) calculated as the mean of the suprabasal 1 and 5 min plasma insulin values. *n *= 11–12 per diet group, * *p* < 0.05, ** *p* < 0.01.

Intravenous glucose tolerance tests were performed after 8 weeks of feeding with the respective diets. Plasma glucose was significantly elevated during the 75 min IVGTT in the high fat diet group compared to the normal diet control, whereas there were no significant differences in plasma glucose levels between the high fat-high sucrose diet and the high sucrose control diet groups (Figure 2A,B). Glucose tolerance, as assessed by the glucose elimination constant KG, was however significantly impaired in both high fat diet groups compared to their respective controls (Figure 2C). Insulin secretion varied significantly between the two diet models (Figure 2D,E). The incremental area under the curve (AUC) for insulin was similar between the normal and high fat diet groups, whereas there was a 2.7 fold increase in AUC in the high fat-high sucrose group compared to the high sucrose control diet group (Figure 2E). The acute insulin response (AIR) to the glucose challenge was not different between the normal diet and the high fat diet groups, but it was significantly higher in the high fat-high sucrose group than in the high sucrose control diet group (Figure 2F). Surprisingly, the high sucrose control diet group had a lower acute insulin response than the normal diet group, although this did not reach statistical significance (*p* = 0.069) (Figure 2F).

**Figure 3:**
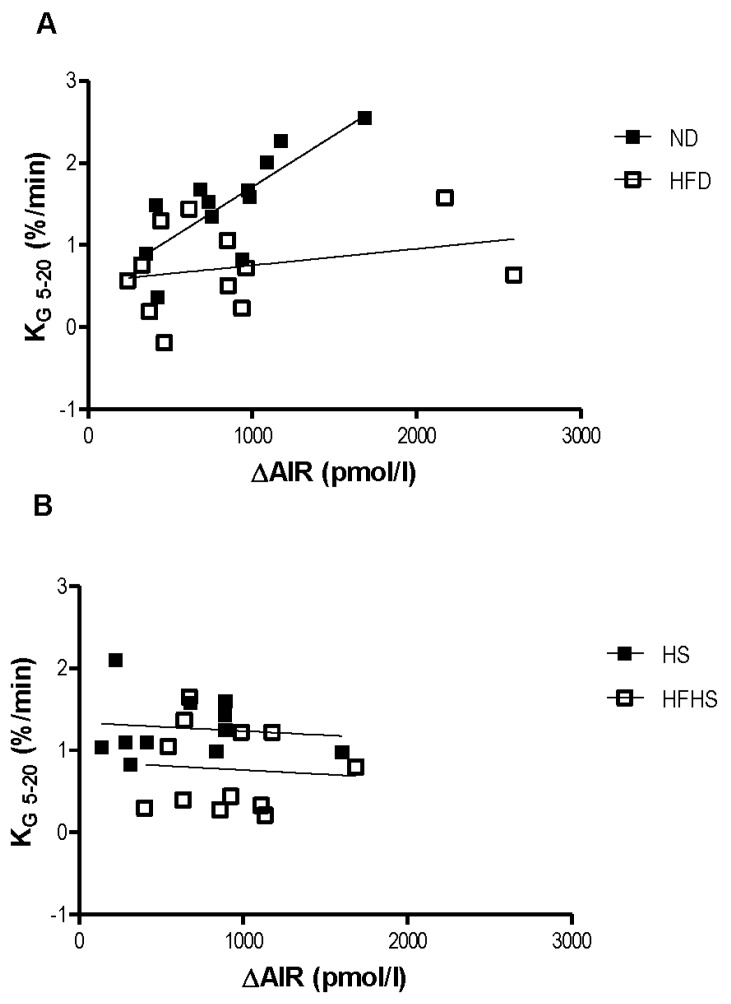
Relationship between insulin secretion and glucose clearance. Linear regressions between the glucose elimination rate (KG) and the acute insulin response (ΔAIR) for the normal and high fat diet groups (**A**) and the Surwit high sucrose and high-fat high sucrose diet groups (**B**). *n* = 11–12 per group.

The relationship between the acute insulin response to the glucose challenge and the rate of glucose elimination was dramatically different between the two diet models. In the normal diet group the rate of glucose elimination (KG) positively correlated with the acute insulin response (Figure 3A). This positive correlation is nearly ablated in the high fat diet group (Figure 3A). Surprisingly, there was no relationship at all between KG and the acute insulin response in either of the high sucrose diet groups (Figure 3B). 

**Figure 4:**
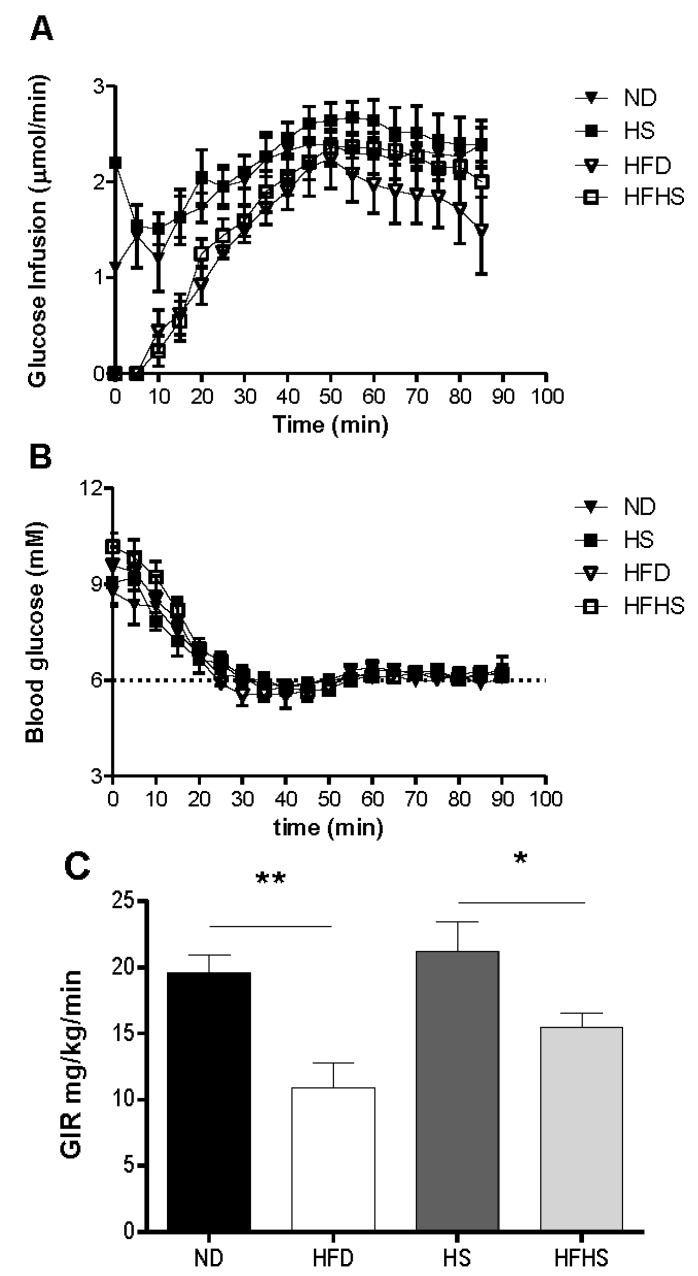
Insulin sensitivity determined by euglycemic-hyperinsulinemic clamp. Anesthetized mice received continuous infusion of insulin (15 mU kg^−1^·min^−1^) and variable infusion of glucose to maintain blood glucose between 5.5 and 6.5 mM. (**A**) The glucose infusion rate for the entire 90 min. (**B**) The blood glucose concentration during the 90 min clamp. (**C**) The mean glucose infusion rate (GIR) for the 60–90 min steady state. *n* = 6–7 per group.

**Figure 5:**
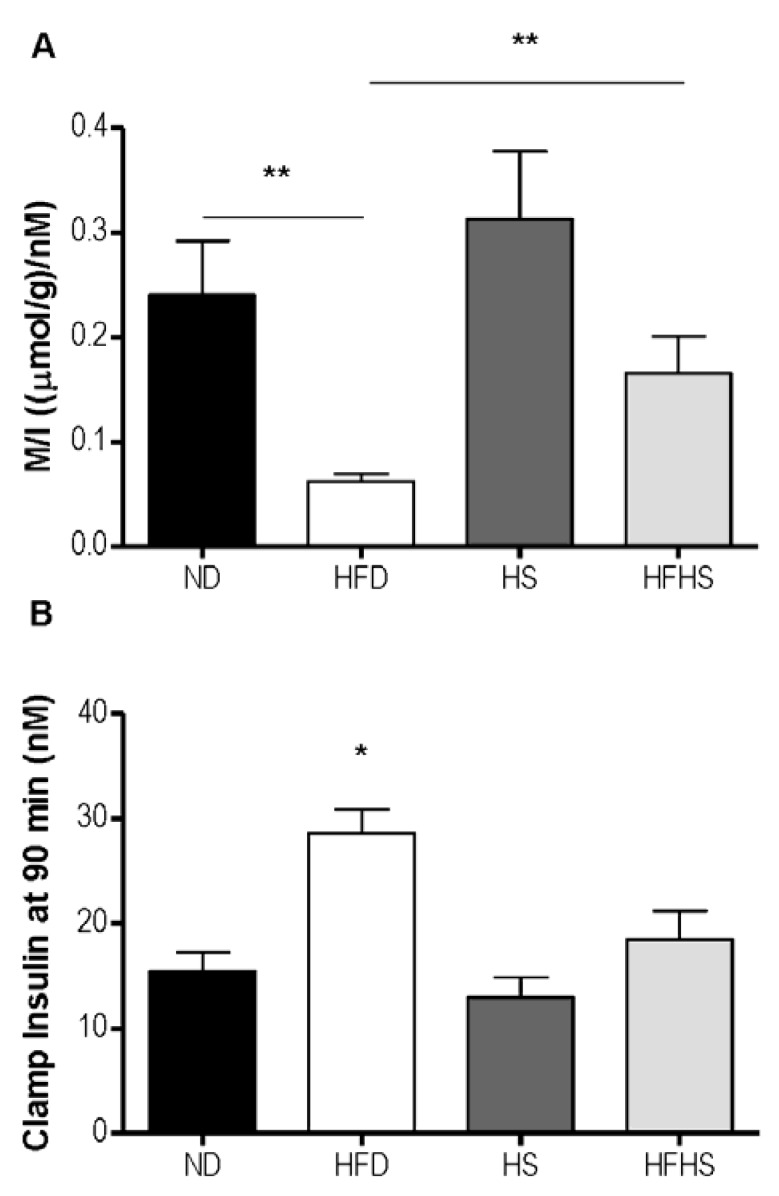
The M/I index derived from the euglycemic-hyperinsulinemic clamp. (**A**) M/I expressed as the ratio of glucose infused (M) to the plasma concentration of insulin (I) at the end of the clamp experiment (90 min). (**B**) The plasma insulin concentration at 90 min. *n* = 6–7 per group, * *p* < 0.05, ** *p* < 0.01.

A most critical property of any diet induced obesity model used to study T2D, is the induction of insulin resistance by the diet. We evaluated this by the euglycemic-hyperinsulinemic clamp, which is the most accurate method for determining insulin sensitivity. Mice that had been fed one of each of the experimental diets were subjected to euglycemic-hyperinsulinemic clamp studies. A variable rate of glucose was infused (Figure 4A) to reach a steady state blood glucose concentration of 6 mM during the final 30 min of the clamp (Figure 4B). The glucose infusion rate for the high fat diet group was 80% lower than that of the normal diet group (Figure 4C). The glucose infusion rate in the high fat-high sucrose diet group however, was only 37% lower than that of the high sucrose control diet group (Figure 4C). Insulin sensitivity can be more accurately assessed by the M/I ratio, which relates the total amount of glucose infused to the final concentration of plasma insulin. The M/I ratio for the high fat diet group was 4-fold lower than that of the normal diet group, whereas in the high fat-high sucrose diet group the M/I ratio was only 1.9 fold lower than the high sucrose control diet group and did not reach statistical significance (Figure 5A). Both of the high sucrose diet groups had slightly higher M/I ratios than their respective low sucrose diet groups, which was significant between diet groups receiving high fat (Figure 5A). The blood insulin level at the end of the 90 min euglycemic-hyperinsulinemic clamp (clamp insulin) was significantly elevated for the HFD group compared to all other groups (Figure 5B).

## 4. Discussion

Diet induced obesity mouse models are used for the development of treatments for many of the co-morbidities of obesity including type 2 diabetes. Two diet induced obesity models frequently used in the study of type 2 diabetes, the 60% high fat diet and the Surwit high fat-high sucrose diet, were evaluated in a head to head comparison of their potential to induce glucose intolerance. The most commonly used mouse strain for the study of diet induced obesity is the C57BL/6 strain, which has been shown to become obese and glucose intolerant after short term feeding with high fat diets [7]. It should be noted that there are many substrains of C57BL/6 and the choice of substrain for this type of study should be carefully considered. The C57BL6/J BomTac line used in this study is derived from the original C57BL6/J line from the Jackson laboratory in Bar Harbor Maine. The C57BL6/J Bom line diverged from the C57BL6/J line in 1971 when it was shipped to Hamburg Germany and the C57BL6/J BomTac line diverged in 1988 when it was sent to Skensved Denmark. At some point between 1976 and 1984 a mutation in the Nicotinamide Nucleotide Transhydrogenase (Nnt) gene arose in the C57BL/6J colony at the Jackson laboratory. This mutation is reported to affect glucose homeostasis and insulin secretion [19]. As the C57BL6/J Bom line was derived at least five years prior to emergence of the mutation, they do not contain the Nnt mutation. This has been confirmed by PCR genotyping. As the point of our study is to determine how the different diets affect beta cell function and adaptation to insulin resistance it is important that there are no genetic factors in the C57BL6 strain used that could affect these parameters. 

There are many differences between the diets including the macronutrient composition. Although the two diet models have nearly identical amounts of dietary kilocalories from fat, the sources of fat differ between all of the diets (Table 2). The normal rodent diet contains approximately equal amounts of kilocalories from animal fat (lard) and vegetable oils (soybean oil). The Surwit diets obtain all of their fat kilocalories from vegetable oils (coconut and soybean oils). This is of relevance for the high fat diets in particular as lard, which is the primary fat source in the high fat diet, has a much different fatty acid composition than coconut oil, the primary fat source in the high fat-high sucrose diet (see Table 2). The different sources of fat are likely the main contributing factor to the differences in weight gain between the two high fat diet models despite having the same energy intake. It has been shown that high fat diets rich in medium chain fatty acids result in less weight gain and adiposity than diets rich in long chain fatty acids. This is due to an increase in fat oxidation and energy expenditure in mice fed the medium chain fatty acid rich diet [20]. The coconut oil based Surwit high fat high sucrose diet has 57% of its fat content from medium chain fatty acids, whereas the lard based high fat diet has virtually no (<0.5%) medium chain fatty acids. The most common fatty acids consumed by humans in a typical “western” diet are long chain fatty acids. In the Surwit high fat-high sucrose diet, 93% of the fat content is from saturated fatty acids. In contrast, the high fat diet obtains 32% of its fat content from saturated fatty acids. Certain fatty acids have been shown to induce lipotoxicity and dysfunction in beta cells [21]. Chronic exposure to palmitate causes diminished glucose stimulated insulin secretion in isolated islets [22]. The standard 60% high fat diet contains more than twice as much palmitate as does the high fat-high sucrose Surwit diet. In contrast the predominant saturated fatty acid in the Surwit high fat-high sucrose diet is laurate which has been shown to have a stimulatory effect on insulin secretion in perifused islets [23].

There were several differences in the insulin secretory response to the glucose challenge in each of the diets. The acute insulin response to glucose was lower in the high sucrose diet group than in any other diet group. This suggests that the relatively high sucrose content of the Surwit control diet has a negative effect on beta cell function independent of dietary fat content. An inhibitory effect of sucrose on the acute insulin response was suggested, but not demonstrated, previously in mice fed high sucrose diets for long periods of time [24]. However in that study oral glucose tolerance tests were used. We decided to use intravenous, as opposed to oral, glucose tolerance tests as intravenous administration of glucose results in direct (<30 s) exposure of the beta cells to supraphysiological concentrations of glucose. This allows for the calculation of the AIR, glucose elimination constant KG and other data points that can be obtained from mathematical modeling of glucose and insulin dynamics. Thus the IVGTT provides powerful tools for determining beta cell function. Oral glucose tolerance tests, while physiological in route of delivery, do not result in direct exposure of the beta cells to glucose as gastric emptying rate, glucose absorption and incretin effects are major factors determining the insulin secretion response to the glucose challenge. 

Sucrose is a disaccharide consisting of a glucose monomer and a fructose monomer connected by glycosidic linkage. Recently, the deleterious effects of a fructose rich diet on islet function were described [25]. Mice fed a high fructose diet for 16 weeks had diminished glucose stimulated insulin secretion *ex-vivo* and increased expression of pro-inflammatory cytokines in islets. The fructose component of sucrose may therefore be a contributing factor to the diminished acute insulin response seen in mice fed a high sucrose diet. The acute insulin response was not different between the two high fat diet models but there were differences in other measures of insulin secretion. Basal insulin was increased in the high fat diet group compared to the control group. Basal insulin in the high fat-high sucrose Surwit diet was not significantly different than in the high sucrose control diet. The increase in basal insulin secretion in response to consumption of a high fat diet in mice corresponds to an increase in beta cell area [11]. It is possible that there is a greater increase in beta cell area in mice fed a high fat diet than that of the high fat-high sucrose diet, although further studies need to be done to confirm this. The high fat-high sucrose diet group had a much higher incremental area under the curve for insulin during the first 50 min of the IVGTT than any of the other diet groups. This difference was primarily the result of the high fat-high sucrose fed group having significantly higher insulin levels after the insulin peak (5, 10, and 20 min). Whether this difference represents increased late stage insulin secretion or decreased insulin clearance in the high-fat diet group can only be definitively determined by C-peptide measurement. However, insulin levels after the euglycemic-hyperinsulinemic clamp were significantly lower in this group; therefore it is unlikely that higher insulin levels during the IVGTT would be the result of decreased insulin clearance. We noted that the high fat-high sucrose group had the highest AUC for insulin secretion even though there was a trend towards a decreased insulin secretion in the high sucrose group. As the HS diet contains 80% kcal from sucrose and the HFHS has 12.5% it is likely that the concentration of sucrose that the islets are exposed to in the HFHS diet is too low have the kind of deleterious effects on insulin secretion that are seen in the HS diet.

One method for evaluating beta cell function in the context of glucose tolerance is to compare the relationship between the acute insulin response to the intravenous glucose load and the corresponding rate of systemic glucose elimination. In mice fed a normal diet increases in the acute insulin response corresponded with increases in the rate of glucose elimination. This relationship was nearly ablated in mice fed a high fat diet as increases in the acute insulin response only resulted in minimal increases to the rate of glucose elimination. Surprisingly, this relationship was not seen at all in either of the high sucrose diets regardless of fat content. Increases in the acute insulin response did not change the rate of glucose elimination, which remained fairly constant across a range of AIR values. This was not a result of insulin resistance as these mice were more insulin sensitive than the high fat diet fed mice, as determined by the euglycemic-hyperinsulinemic clamp. The relationship between glucose elimination and insulin secretion in the high sucrose diet models will be examined in more detail in subsequent studies.

When evaluating diet-induced obesity models for the study of type 2 diabetes a most important factor for the model is the robust induction of insulin resistance. The euglycemic-hyperinsulinemic clamp was used to determine insulin sensitivity in the diet models, as in previous studies we have shown that high fat diet fed mice have insulin resistance as determined by this technique [18]. We confirmed now that insulin resistance, as determined by the euglycemic clamp, was robustly induced after 8 weeks of feeding the high fat diet. In contrast, we found that there was a much more modest induction of insulin resistance with the high fat-high sucrose diet after 8 weeks. The M/I ratio was thus significantly higher in the high fat-high sucrose diet group than in the high fat diet group. This difference between the two diets, the mechanism of which needs to be explored in further studies, is important to acknowledge when selecting diet for experimental studies in obesity and diabetes. 

## 5. Conclusion

In conclusion, our results give a clear indication that the different macronutrient composition of the two most commonly used high fat diets result in different models with respect to the degree of insulin resistance and beta cell dysfunction induced in a relatively short period of time. Of particular importance is a clear difference in degree of insulin resistance induced by high-fat *versus* high fat-high sucrose diets. Furthermore, the high sucrose control diet imparts a unique phenotype with beta cell dysfunction present already in lean, insulin sensitive animals. Further studies will need to be done to determine how these phenotypes develop over time and to determine the molecular mechanisms responsible for the differences in insulin secretion and beta cell function between the different die models.

## References

[1] Wild S., Roglic G., Green A., Sicree R., King H. (2004). Global prevalence of diabetes: Estimates for the year 2000 and projections for 2030. Diabetes Care.

[2] Guh D.P., Zhang W., Bansback N., Amarsi Z., Birmingham C.L., Anis A.H. (2009). The incidence of co-morbidities related to obesity and overweight: A systematic review and meta-analysis. BMC Public Health.

[3] Boden G., Shulman G.I. (2002). Free fatty acids in obesity and type 2 diabetes: Defining their role in the development of insulin resistance and beta-cell dysfunction. Eur. J. Clin. Invest..

[4] Kahn S.E. (2003). The relative contributions of insulin resistance and beta-cell dysfunction to the pathophysiology of type 2 diabetes. Diabetologia.

[5] Ritzel R.A., Butler A.E., Rizza R.A., Veldhuis J.D., Butler P.C. (2006). Relationship between beta-cell mass and fasting blood glucose concentration in humans. Diabetes Care.

[6] Dunning B.E., Foley J.E., Ahren B. (2005). Alpha cell function in health and disease: Influence of glucagon-like peptide-1. Diabetologia.

[7] Surwit R.S., Kuhn C.M., Cochrane C., McCubbin J.A., Feinglos M.N. (1988). Diet-induced type 2 diabetes in C57BL/6J mice. Diabetes.

[8] Winzell M.S., Ahren B. (2004). The high-fat diet-fed mouse: A model for studying mechanisms and treatment of impaired glucose tolerance and type 2 diabetes. Diabetes.

[9] Winzell M.S., Magnusson C., Ahren B. (2007). Temporal and dietary fat content-dependent islet adaptation to high-fat feeding-induced glucose intolerance in mice. Metabolism.

[10] Hull R.L., Kodama K., Utzschneider K.M., Carr D.B., Prigeon R.L., Kahn S.E. (2005). Dietary-fat-induced obesity in mice results in beta cell hyperplasia but not increased insulin release: Evidence for specificity of impaired beta cell adaptation. Diabetologia.

[11] Ahren J., Ahren B., Wierup N. (2010). Increased beta-cell volume in mice fed a high-fat diet: A dynamic study over 12 months. Islets.

[12] Talchai C., Lin H.V., Kitamura T., Accili D. (2009). Genetic and biochemical pathways of beta-cell failure in type 2 diabetes. Diabetes Obes. Metab..

[13] Butler A.E., Janson J., Bonner-Weir S., Ritzel R., Rizza R.A., Butler P.C. (2003). Beta-cell deficit and increased beta-cell apoptosis in humans with type 2 diabetes. Diabetes.

[14] Rahier J., Guiot Y., Goebbels R.M., Sempoux C., Henquin J.C. (2008). Pancreatic beta-cell mass in european subjects with type 2 diabetes. Diabetes Obes. Metab..

[15] Bjursell M., Gerdin A.K., Lelliott C.J., Egecioglu E., Elmgren A., Tornell J., Oscarsson J., Bohlooly Y.M. (2008). Acutely reduced locomotor activity is a major contributor to western diet-induced obesity in mice. Am. J. Physiol. Endocrinol. Metab..

[16] Gault V.A., McClean P.L., Cassidy R.S., Irwin N., Flatt P.R. (2007). Chemical gastric inhibitory polypeptide receptor antagonism protects against obesity, insulin resistance, glucose intolerance and associated disturbances in mice fed high-fat and cafeteria diets. Diabetologia.

[17] Surwit R.S., Feinglos M.N., Rodin J., Sutherland A., Petro A.E., Opara E.C., Kuhn C.M., Rebuffe-Scrive M. (1995). Differential effects of fat and sucrose on the development of obesity and diabetes in C57BL/6J and A/J mice. Metabolism.

[18] Pacini G., Thomaseth K., Ahren B. (2001). Contribution to glucose tolerance of insulin-independent *vs. *insulin-dependent mechanisms in mice. Am. J. Physiol. Endocrinol. Metab..

[19] Toye A.A., Lippiat J.D., Proks P., Shimomura K., Bentley L., Hugill A., Mijat V., Goldsworthy M., Moir L., Haynes A. (2005). A genetic and physiological study of impaired glucose homeostasis control in C57BL/6J mice. Diabetologia.

[20] De Vogel-van den Bosch J., van den Berg S.A., Bijland S., Voshol P.J., Havekes L.M., Romijn H.A., Hoeks J., van Beurden D., Hesselink M.K., Schrauwen P. (2011). High-fat diets rich in medium- *vs.* long-chain fatty acids induce distinct patterns of tissue specific insulin resistance. J. Nutr. Biochem..

[21] Wang Y., Wang P.Y., Takashi K. (2006). Chronic effects of different non-esterified fatty acids on pancreatic islets of rats. Endocrine.

[22] Hoppa M.B., Collins S., Ramracheya R., Hodson L., Amisten S., Zhang Q., Johnson P., Ashcroft F.M., Rorsman P. (2009). Chronic palmitate exposure inhibits insulin secretion by dissociation of Ca^2+^ channels from secretory granules. Cell Metab..

[23] Lee S.K., Opara E.C., Surwit R.S., Feinglos M.N., Akwari O.E. (1995). Defective glucose-stimulated insulin release from perifused islets of C57BL/6J mice. Pancreas.

[24] Sumiyoshi M., Sakanaka M., Kimura Y. (2006). Chronic intake of high-fat and high-sucrose diets differentially affects glucose intolerance in mice. J. Nutr..

[25] Caton P.W., Kieswich J., Yaqoob M.M., Holness M.J., Sugden M.C. (2011). Nicotinamide mononucleotide protects against pro-inflammatory cytokine-mediated impairment of mouse islet function. Diabetologia.

